# Boundary value problems for hypergenic function vectors

**DOI:** 10.1186/s13660-018-1725-8

**Published:** 2018-06-13

**Authors:** Guiling Zhang, Chong Li, Yonghong Xie

**Affiliations:** College of Mathematics and Information Science, Hebei Normal University, Shijiazhuang, P.R. China

**Keywords:** Clifford analysis, Hypergenic function vector, Nonlinear boundary value problem, Linear boundary value problem

## Abstract

This article mainly studies the boundary value problems for hypergenic function vectors in Clifford analysis. Firstly, some properties of hypergenic quasi-Cauchy type integrals are discussed. Then, by the Schauder fixed point theorem the existence of the solution to the nonlinear boundary value problem is proved. Finally, using the compression mapping principle the existence and uniqueness of the solution to the linear boundary value problem are proved.

## Introduction

A Clifford algebra is an associative and noncommutable algebra [1]. In 1982, Brackx, Delanghe and Sommen [2] established the theoretical basis of Clifford analysis. In recent years, Clifford analysis has been widely used in physics and in mathematics [3–5]. Eriksson [6–8], Huang [9, 10], Qiao [11, 12], Xie [13–17] and Yang [18, 19] have done a lot of work in Clifford analysis. In 1996, Huang [10] studied the nonlinear boundary value problem for biregular functions in Clifford analysis. In 2000, Cai, Huang and Qiao [20] studied the nonlinear boundary value problem for biregular functions vector in Clifford analysis. In 2003, Xie, Qiao and Jiao [20] studied a nonlinear boundary value problem for a generalized biregular function vector. In 2005, Qiao [11] discussed a linear boundary value problem for hypermonogenic functions in Clifford analysis. In 2009–2010, Eriksson and Orelma [6, 7] studied hypergenic functions in the real Clifford algebra $\mathit {Cl}_{n+1,0}(\mathbb{R})$ and its Cauchy integral formula was given. In 2014, Xie [14, 15] studied the Cauchy integral for dual *k*-hypergenic functions and the boundary properties of the hypergenic quasi-Cauchy integral in real Clifford analysis were given. In 2016, Xie, Zhang and Tang [17] discussed some properties of *k*-hypergenic functions.

On the basis of the above, the boundary value problems for hypergenic function vectors are proved.

## Preliminaries

See [6]; let $\mathit {Cl}_{n+1,0}(\mathbb{R})$ be a real Clifford algebra and have identity element $e_{\varnothing}=1$ and basis elements $e_{0}, e_{1},\ldots, e_{n};e_{0}e_{1},\ldots,e_{n-1}e_{n}; \ldots ;e_{0}e_{1}\cdots e_{n}$, and satisfy

$$\textstyle\begin{cases} e_{i}e_{j} = -e_{j}e_{i},\quad i\ne j, i,j=0,1,\ldots,n; \\ e^{2}_{j} = +1,\quad j=0,1,\ldots,n. \end{cases} $$

Any element in $\mathit {Cl}_{n+1,0}(\mathbb{R})$ has the form $a=\sum_{A}a_{A}e_{A}$, $e_{A}=e_{\alpha_{1}}e_{\alpha_{2}}\cdots e_{\alpha _{h}}$ or $e_{\varnothing}=1$, where $A=\{\alpha_{1},\alpha_{2},\ldots,\alpha_{h} \}$, $0\le \alpha_{1}<\alpha_{2}<\cdots<\alpha_{h}\le n$, $a_{A}\in\mathbb {R}$. The norm of $a\in \mathit {Cl}_{n+1,0}(\mathbb{R})$ is defined as $\vert a \vert ={(\sum_{A} \vert a_{A} \vert ^{2})}^{\frac{1}{2}}$. In this paper $J_{i}$ ($i=1,2,\ldots, 32$) is a positive constant. For any $a,b\in{ \mathit {Cl}_{n+1,0}(\mathbb{R})}$, we have

1 $$ \vert {a+b} \vert \leq \vert {a} \vert + \vert {b} \vert ,\quad\quad \vert {ab} \vert \leq{J_{1}} \vert {a} \vert \vert {b} \vert . $$

If $a=a_{0}e_{0}+a_{1}e_{1}+\cdots+a_{n}e_{n}$, it may be observed that $a^{2}= \vert a \vert ^{2}$ and when $a\neq0$ the inverse of *a* is $a^{-1}= {\frac{a}{ \vert a \vert ^{2}}}$. See [6]; any element $a\in \mathit {Cl}_{n+1,0}(\mathbb {R})$ can be uniquely decomposed as $a=b+e_{0}c$, where $b, c\in \mathit {Cl}_{n,0}(\mathbb{R})$. As regards decomposition we can define the mappings $P_{0}:\mathit {Cl}_{n+1,0}\rightarrow \mathit {Cl}_{n,0}$ and $Q_{0}: \mathit {Cl}_{n+1,0}\rightarrow \mathit {Cl}_{n,0}$ by $P_{0}a=b$, $Q_{0}a=c$, where *b*, *c* are called the $P_{0}$ part and the $Q_{0}$ part of *a*, respectively.

Let $\Omega_{0}$ be a nonempty open connected set in ${R^{n+1}}$. The function $f:\Omega_{0}\rightarrow \mathit {Cl}_{n+1,0}(\mathbb{R})$ is denoted by $f(x)=\sum_{A}f_{A}(x)e_{A}$, where $f_{A}\in\mathbb{R}$. The function $f:\Omega_{0}\rightarrow \mathit {Cl}_{n+1,0}(\mathbb{R})$ is said to be continuous on ${\Omega_{0}}$ if and only if each component $f_{A}(x)$ of $f(x)$ is continuous on ${\Omega_{0}}$. Suppose ${C^{r}(\Omega_{0},\mathit {Cl}_{n+1,0}(\mathbb{R}))}=\{f\mid f:\Omega _{0}\rightarrow \mathit {Cl}_{n+1,0}(\mathbb{R}),f(x)=\sum_{A}f_{A}(x)e_{A}$, where $f_{A}$ is *r*-times continuously differentiable on $\Omega_{0}$ and $r\in\mathbb{N}^{*}\}$.

For $f\in{C^{1}(\Omega_{0},\mathit {Cl}_{n+1,0}(\mathbb{R}))}$, we introduce Dirac operators as follows [6]:

$$Df=\sum_{l=0}^{n}e_{l} \frac{\partial{f}}{\partial{x_{l}}}. $$

### Definition 2.1

([15])

A Lyapunov surface *S* is a surface satisfying the following three conditions:

1. Through each point in *S*, there is a tangent plane.
2. There is a real constant number *d* such that, for any $N_{0}\in {S}$, *E* is a ball with radius *d*, centered at $N_{0}$, and *E* is divided into two parts by *S*, the part of *S* lying in the interior of *E* is denoted by $S^{\prime}$, the other is in the exterior of *S*: and each straight line parallel to the normal direction of *S* at $N_{0}$ intersects it at one point.
3. If the angle $\theta(N_{1},N_{2})$ between outward normal vectors through $N_{1}$, $N_{2}$ is an acute angle and *r* is the distance between $N_{1}$ and $N_{2}$, then there are two numbers *β*, *α* ($0\leq\alpha\leq1$, $\beta>0$) independent of $N_{1}$, $N_{2}$ such that $\theta(N_{1},N_{2})\leq\beta{r}^{\alpha}$.

### Definition 2.2

([15])

The function *f*: $\partial\Omega_{0}\longrightarrow \mathit {Cl}_{n+1,0}(\mathbb{R})$ is said to be Hölder continuous on $\Omega _{0}$ if there exists a positive constant $M_{0}$ such that $\vert f(x_{1})-f(x_{2}) \vert \leq{M_{0} \vert x_{1}-x_{2} \vert ^{\beta}}$ ($0<\beta<1$) holds for any $x_{1},x_{2}\in\partial\Omega_{0}$.

The set of all Hölder continuous functions which are defined on $\Omega_{0}$ and valued in $\mathit {Cl}_{n+1,0}(\mathbb{R})$ is denoted by $H(\beta,\partial\Omega_{0},\mathit {Cl}_{n+1,0}(\mathbb{R}))$.

For any $f\in{H(\beta,\partial\Omega_{0},\mathit {Cl}_{n+1,0}(\mathbb{R}))}$, we define the norm of *f* as ${ \Vert f \Vert _{\beta }}=C(f,\partial\Omega _{0})+H(f,\partial\Omega_{0},\beta)$, where

$${C(f,\partial\Omega_{0})}=\max_{t\in\partial\Omega_{0}} \bigl\vert f(t) \bigr\vert , \quad\quad{H(f,\partial\Omega_{0}, \beta)}=\sup_{\substack{t_{1}\neq{t_{2}} \\ t_{1},t_{2}\in{\partial\Omega_{0}}}} {\frac { \vert f(t_{1})-f(t_{2}) \vert }{ \vert t_{1}-t_{2} \vert ^{\beta}}} . $$

It is easy to prove that $H(\beta,\partial\Omega _{0},\mathit {Cl}_{n+1,0}(\mathbb{R}))$ forms a Banach space.

For any ${f,g}\in{H(\beta,\partial\Omega_{0},\mathit {Cl}_{n+1,0}(\mathbb {R}))}$, we have

2 $$ \Vert {f+g} \Vert _{\beta}\leq{{ \Vert {f} \Vert _{\beta }}+{ \Vert {g} \Vert _{\beta}}}, \quad\quad { \Vert {fg} \Vert _{\beta}}\leq{{J_{2}} {{ \Vert {f} \Vert _{\beta}} { \Vert {g} \Vert _{\beta}}}}. $$

In this paper, let Ω be a domain in $\mathbb{R}^{n+1}_{+}={\{ x\mid (x_{0},x_{1},\ldots,{x_{n}})\in\mathbb{R}^{n+1},x_{0}>0\}}$, and its boundary *∂*Ω be a smooth compact oriented Lyapunov surface. For any $f\in C^{1}(\Omega, \mathit {Cl}_{n+1,0}(\mathbb{R}))$, we introduce a modified Dirac operator as follows [6]:

$$Hf=Df- \frac{n-1}{x_{0}}{Q_{0}}f. $$

### Definition 2.3

([6])

$f(x)$ is said to be a hypergenic function on Ω if $f\in {C^{1}(\Omega, \mathit {Cl}_{n+1,0}(\mathbb{R}))}$ satisfies $Hf=0$ on Ω.

In this paper, let $E_{1}(x,y)= {\frac{x-y}{ \vert x-y \vert ^{n+1} \vert x-\widehat {y} \vert ^{n-1}}}$, $E_{2}(x,y)= {\frac{\widehat{x}-y}{ \vert x-y \vert ^{n-1} \vert x-\widehat {y} \vert ^{n+1}}}$, and $w_{n+1}$ is the surface area of the unit hypersphere in $\mathbb{R}^{n+1}$.

### Definition 2.4

([15])

3 $$ \Psi_{f}(y)={ \frac{{2^{n-1}}{y_{0}^{n-1}}}{w_{n+1}}} { \biggl[ \int_{\partial\Omega}{E_{1}(x,y)\,d\sigma(x)f(x)}}-{ \int_{\partial \Omega}{E_{2}(x,y)\widehat{d\sigma(x)} \widehat{f(x)}} \biggr]} $$

is called a hypergenic quasi-Cauchy type integral if $f\in{H(\beta ,\partial\Omega, \mathit {Cl}_{n+1,0}(\mathbb{R}))}$.

### Lemma 2.1

([14])

*If* $y\notin{\partial\Omega}$, $f\in{H(\beta,\partial\Omega ,\mathit {Cl}_{n+1,0}(\mathbb{R}))}$, *the hypergenic quasi*-*Cauchy type integral*

$${\Psi_{f}{(y)}={\frac{{2^{n-1}}{y_{0}^{n-1}}}{w_{n+1}}} { \biggl[ \int_{\partial\Omega}{E_{1}(x,y)\,d\sigma(x)f(x)}}-{ \int_{\partial\Omega }{E_{2}(x,y)\widehat{d\sigma(x)} \widehat{f(x)}} \biggr]}} $$

*is a hypergenic function on* ${{\mathbb{R}}_{+}^{n+1}}\backslash {{\partial\Omega}}$.

### Remark 2.1

If $y\notin{\partial\Omega}$, $f\in{H(\beta,\partial\Omega ,\mathit {Cl}_{n+1,0}(\mathbb{R}))}$, the hypergenic quasi-Cauchy type integral

$${\Psi_{f}{(y)}={\frac{{2^{n-1}}{y_{0}^{n-1}}}{w_{n+1}}} { \biggl[ \int_{\partial\Omega}{E_{1}(x,y)\,d\sigma(x)f(x)}}-{ \int_{\partial\Omega }{E_{2}(x,y)\widehat{d\sigma(x)} \widehat{f(x)}} \biggr]}} $$

satisfies $\Psi_{f}{(\infty)}=0$.

### Lemma 2.2

([15])

*If* $f\in{H(\beta,\partial\Omega, \mathit {Cl}_{n+1,0}(\mathbb{R}))}$, *then* $\Psi_{f}(y)$ *is Hölder continuous on* $\Omega^{+}\cup\partial \Omega$ *and* $\Omega^{-}\cup\partial\Omega$.

Let $\mathbf{B}(\mathbf{y},\delta)$ be a ball with radius $\delta>{0}$, centered at *y* when $y\in{\partial\Omega}$. *∂*Ω is divided into two parts by $\mathbf{B}(\mathbf{y},\delta)$. The part of *∂*Ω lying in the interior of $\mathbf{B}(\mathbf{y},\delta)$ is denoted by $\lambda_{\delta}$.

### Definition 2.5

([15])

**I** is called the Cauchy principal of the singular integral value if $\lim_{\delta\rightarrow{0}}\Psi_{f}(y)=\mathbf{I}$ exists, and we write directly $\mathbf{I}=\Phi_{f}(y)$.

### Lemma 2.3

([15])

*If* $y\in\partial\Omega$, $f\in{H(\beta,\partial\Omega ,\mathit {Cl}_{n+1,0}(\mathbb{R}))}$, *then the Cauchy principal values of the singular integral* (3) *exist*, *and*

4 $$ \begin{aligned}[b] \Phi_{f}(y)& ={ \frac {{2^{n-1}}{y_{0}^{n-1}}}{w_{n+1}}} { \biggl[ \int_{\partial\Omega}{E_{1}(x,y)\,d\sigma(x) \bigl(f(x)-f(y) \bigr)}} \\ &\quad{} -{ \int_{\partial\Omega}{E_{2}(x,y)\widehat{d\sigma(x)} \bigl( \widehat{f(x)}-f(y) \bigr)} \biggr]}+ \frac{1}{2}f(y), \end{aligned} $$

*when* $f=1$, *we have* $\Phi_{1}(y)= \frac{1}{2}$.

### Lemma 2.4

([15])

*If* $y\in{\partial\Omega}$, $f\in{H(\beta,\partial\Omega ,\mathit {Cl}_{n+1,0}(\mathbb{R})})$, *then*

5 $$ \textstyle\begin{cases} \Psi^{+}_{f}(y)=\Phi_{f}(y)+ \frac{1}{2}f(y), \\ \Psi^{-}_{f}(y)=\Phi_{f}(y)- \frac{1}{2}f(y). \end{cases} $$

### Lemma 2.5

([5])

*If* Ω *is a bounded domain in* $\mathbb{R}^{n+1}_{+}$, $0<\alpha <n+1$, *for any* $y\in\Omega$ *we have*

$$\int_{\Omega} \vert x-y \vert ^{-\alpha}\,dx \leq{M_{1}}(\alpha,\Omega), $$

*where* ${M_{1}}(\alpha,\Omega)$ *is a positive constant only related to* *α*, Ω.

### Definition 2.6

$F(x)=(f_{1}(x),\ldots,f_{q}(x))$ is called a function vector if $f_{i}(x):\Omega\rightarrow \mathit {Cl}_{n+1,0}(\mathbb{R})$ ($i=1,\ldots,q$).

For $F(x)=(f_{1}(x),\ldots,f_{q}(x))$, $K(x)=(k_{1}(x),\ldots ,k_{q}(x))$, define the addition operation and multiplication operation for function vectors as follows:

$$F\oplus K =(f_{1}+k_{1},\ldots,f_{q}+k_{q});F \otimes K =(f_{1}k_{1},\ldots,f_{q}k_{q}). $$

Let $L(x)$ be a function valued in Clifford algebra $\mathit {Cl}_{n+1,0}(\mathbb {R})$ and $F(x)$ be a function vector, then

$$LF=(Lf_{1},\ldots,Lf_{q}),\quad\quad FL=(f_{1}L,\ldots,f_{q}L). $$

Define the model of a function vector as follows: $\vert F(x) \vert =(\sum_{i=0}^{q} \vert f_{i}(x) \vert ^{2})^{\frac {1}{2}}$, we have

6 $$ \vert F\oplus K \vert \leq \vert F \vert + \vert K \vert , \quad\quad \vert F\otimes K \vert \leq J_{1} \vert F \vert \vert K \vert . . $$

### Definition 2.7

$F(x)=(f_{1}(x),\ldots,f_{q}(x))$ is called a hypergenic function vector when each component $f_{i}(x)$ ($i=1,\ldots,q$) is a hypergenic function on Ω.

### Definition 2.8

A hypergenic function vector *F* is said to be Hölder continuous on *∂*Ω if there is a positive constant $M_{2}$ such that

$$\bigl\vert F(x_{1})-F(x_{2}) \bigr\vert = \Biggl(\sum _{i=0}^{q} \bigl\vert f_{i}(x_{1})-f_{i}(x_{2}) \bigr\vert ^{2} \Biggr)^{\frac{1}{2}}\leq M_{2} \vert x_{1}-x_{2} \vert ^{\beta} $$

holds for any $x_{1},x_{2}\in\Omega$, where $0<\beta<1$ and $M_{2} $ is independent of $x_{i}$ ($i=1,2$).

### Remark 2.2

The hypergenic function vector $F(x)=(f_{1}(x),\ldots,f_{q}(x))$ is Hölder continuous on *∂*Ω if and only if each component $f_{i}(x)$ ($i=1,\ldots,q$) of $F(x)$ is Hölder continuous on *∂*Ω.

The set of all Hölder continuous function vectors which are defined on *∂*Ω and valued in $\mathit {Cl}_{n+1,0}(\mathbb{R})$ is denoted by $H_{q}(\beta,\partial\Omega, \mathit {Cl}_{n+1,0}(\mathbb{R}))$. For any $F\in{H_{q}(\beta,\partial\Omega, \mathit {Cl}_{n+1,0}(\mathbb{R}))}$, the norm of *F* is defined as follows: ${ \Vert F \Vert _{\beta }}=C_{q}(F,\partial\Omega )+H_{q}(F,\partial\Omega,\beta)$, where

$${C_{q}(F,\partial\Omega)}=\max_{t\in\partial\Omega} \bigl\vert F(t) \bigr\vert ,\quad\quad{H_{q}(F,\partial\Omega, \beta)}=\sup_{\substack{t_{1}\neq{t_{2}} \\ t_{1},t_{2}\in{\partial\Omega}}} {\frac { \vert f(t_{1})-f(t_{2}) \vert }{ \vert t_{1}-t_{2} \vert ^{\beta}}} . $$

It is easy to prove that $H_{q}(\beta,\partial\Omega ,\mathit {Cl}_{n+1,0}(\mathbb{R}))$ forms a Banach space.

For any ${F,K}\in{H_{q}(\beta,\partial\Omega, \mathit {Cl}_{n+1,0}(\mathbb {R}))}$, we have

7 $$ \Vert {F+K} \Vert _{\beta}\leq{{ \Vert {F} \Vert _{\beta }}+{ \Vert {K} \Vert _{\beta}}}, \quad\quad{ \Vert {F \otimes K} \Vert _{\beta}}\leq{{J_{3}} {{ \Vert {F} \Vert _{\beta }} { \Vert {K} \Vert _{\beta}}}} . $$

## Some properties of hypergenic quasi-Cauchy type integrals

### Theorem 3.1

*If* $y\notin{\partial\Omega}$, $F(x)=(f_{1}(x),\ldots,f_{q}(x))\in {H_{q}(\beta,\partial\Omega, \mathit {Cl}_{n+1,0}(\mathbb{R}))}$,

8 $$ \Psi_{F}(y)={ \frac {{2^{n-1}}{y_{0}^{n-1}}}{w_{n+1}}} { \biggl[ \int_{\partial\Omega }{E_{1}(x,y)\,d\sigma(x)F(x)}} -{ \int_{\partial\Omega}{E_{2}(x,y)\widehat{d\sigma(x)}\widehat {F(x)}} \biggr]} $$

*is a hypergenic function vector on* ${{\mathbb{R}}_{+}^{n+1}}\backslash {{\partial\Omega}}$, $\Psi_{F}{(\infty)}=0$, *and* $\Psi_{F}(y)$ *is Hölder continuous on* $\Omega^{\pm}\cup\partial\Omega$.

### Proof

$$\begin{aligned} \Psi_{F}(y)&= \biggl(\frac{{2^{n-1}}{y_{0}^{n-1}}}{w_{n+1}} \biggl[ \int_{\partial\Omega}{E_{1}(x,y)\,d\sigma(x)f_{1}(x)}- \int_{\partial\Omega}{E_{2}(x,y)\widehat{d\sigma(x)}\widehat {f_{1}(x)}} \biggr], \\ &\quad\ \cdots, {\frac{{2^{n-1}}{y_{0}^{n-1}}}{w_{n+1}}} \biggl[ \int_{\partial \Omega}{E_{1}(x,y)\,d\sigma(x)f_{q}(x)}- \int_{\partial\Omega }{E_{2}(x,y)\widehat{d\sigma(x)} \widehat{f_{q}(x)}} \biggr] \biggr) \\ &= \bigl(\Psi_{f_{1}}(y),\ldots,\Psi_{f_{q}}(y) \bigr). \end{aligned} $$

It follows from $F(x)=(f_{1}(x),\ldots,f_{q}(x))\in H_{q}(\beta ,\partial\Omega, \mathit {Cl}_{n+1,0}(\mathbb{R}))$ that $f_{i}\in H_{q}(\beta ,\partial\Omega, \mathit {Cl}_{n+1,0}(\mathbb{R}))$ ($i=1,\ldots,q$). From Lemma 2.1, $\Psi_{f_{i}}(y)$ ($i=1,\ldots,q$) is a hypergenic function on $R^{n+1}_{+}\setminus\partial\Omega$. Hence $\Psi_{F}(y)$ is a hypergenic function vector on $R^{n+1}_{+}\setminus\partial\Omega$. By Lemma 2.2 and Remark 2.2 $\Psi_{F}(y)$ is Hölder continuous on $\Omega^{\pm}\cup \partial\Omega$. By Remark 2.1 we conclude $\Psi_{f_{i}}{(\infty)}=0$ ($i=1,\ldots,q$), thus $\Psi_{F}{(\infty)}=0$. □

### Theorem 3.2

*If* $y\in\partial\Omega_{,} F\in{H_{q}(\beta,\partial\Omega ,\mathit {Cl}_{n+1,0}(\mathbb{R})})$, *then*

9 $$ \begin{aligned}[b] \Phi_{F}(y)&={ \frac {{2^{n-1}}{y_{0}^{n-1}}}{w_{n+1}}} { \biggl[ \int_{\partial\Omega}{E_{1}(x,y)\,d\sigma(x) \bigl(F(x)-F(y) \bigr)}} \\ &\quad{} -{ \int_{\partial\Omega}{E_{2}(x,y)\widehat{d\sigma(x)} \bigl( \widehat{F(x)}-F(y) \bigr)} \biggr]}+ \frac{1}{2}F(y). \end{aligned} $$

From Lemma 2.4 we conclude to the following theorem.

### Theorem 3.3

10 $$ \textstyle\begin{cases} \Psi^{+}_{F}(y)=\Phi_{F}(y)+ \frac{1}{2}F(y), \\ \Psi^{-}_{F}(y)=\Phi_{F}(y)- \frac{1}{2}F(y). \end{cases} $$

### Lemma 3.1

([15])

*If* $x,y\in\mathbb{R}^{n+1}$ ($n\geq2$), *m* (≥0) *is an integer*, *then*

$$\biggl\vert \frac{x}{ \vert x \vert ^{m+2}}-\frac{y}{ \vert y \vert ^{m+2}} \biggr\vert \leq \frac {P_{m}(x,y)}{ \vert x \vert ^{m+1} \vert y \vert ^{m+1}} \vert x-y \vert , $$

*where*

$$P_{m}(x,y)= \textstyle\begin{cases} \sum_{l=0}^{m} \vert x \vert ^{m-l} \vert y \vert ^{l},& m\neq0;\\ 1,& m=0. \end{cases} $$

### Theorem 3.4

*If* $y\in{\partial\Omega}$, $F\in{H_{q}(\beta,\partial\Omega, \mathit {Cl}_{n+1,0}(\mathbb{R})})$, $Q(y)= \frac{1}{2}F(y)-\Phi_{F}(y)$, *then*

$$\bigl\Vert {Q}(y) \bigr\Vert _{\beta}\leq{J_{31} \Vert {F} \Vert _{\beta}}. $$

### Proof

Similar to Ref. [15], we have

$$\bigl\vert d\sigma(x) \bigr\vert \leq{M_{3}}\rho^{n-1}\,d \rho, $$

where $M_{3}$ is a positive constant.

From Theorem 3.2, Lemma 2.3 and Lemma 2.4, we get

$$\begin{aligned} & \bigl\vert {Q(y)} \bigr\vert \\ &\quad= \biggl\vert {{ {\frac{{2^{n-1}}{y_{0}^{n-1}}}{w_{n+1}}}} { \biggl[ \int_{\partial\Omega}{E_{1}(x,y)\,d\sigma(x)F(y)}- \int_{\partial \Omega}{E_{2}(x,y)\widehat{d\sigma(x)}F(y)} \biggr]}} \\ & \quad\quad {} - { {\frac{{2^{n-1}}{y_{0}^{n-1}}}{w_{n+1}}}} { \biggl[ \int_{\partial\Omega}{E_{1}(x,y)\,d\sigma(x)F(x)}- \int_{\partial\Omega }{E_{2}(x,y)\widehat{d\sigma(x)} \widehat{F(x)}} \biggr]} \biggr\vert \\ &\quad\leq\biggl\vert { {\frac{{2^{n-1}}{y_{0}^{n-1}}}{w_{n+1}}}} \biggr\vert \biggl[ \biggl\vert { \int_{\partial\Omega}{E_{1}(x,y)\,d\sigma(x) \bigl(F(y)-F(x) \bigr)}} \biggr\vert + \biggl\vert { \int_{\partial\Omega }{E_{2}(x,y)\widehat{d\sigma(x)} \bigl( \widehat{F(x)}-F(y) \bigr)}} \biggr\vert \biggr] \\ &\quad\leq J_{4}H_{q}(F,\partial\Omega,\beta) \int_{\partial \Omega} \bigl\vert E_{1}(x,y) \bigr\vert \bigl\vert \,d\sigma(x) \bigr\vert \vert {y-x} \vert ^{\beta}+ 2\max _{x\in\partial\Omega} \bigl\vert {F(x)} \bigr\vert \int_{\partial\Omega } \bigl\vert E_{2}(x,y) \bigr\vert \bigl\vert \widehat{d\sigma(x)} \bigr\vert \\ &\quad\leq J_{5}H_{q}(F,\partial\Omega,\beta) \int_{\partial \Omega} {\frac{1}{ \vert {x-y} \vert ^{n}}} \bigl\vert {d\sigma{(x)}} \bigr\vert \vert {y-x} \vert ^{\beta }+2\max_{x\in\partial\Omega} \bigl\vert {F(x)} \bigr\vert \int_{\partial\Omega} {\frac {1}{ \vert {x-y} \vert ^{n-1}}} \bigl\vert {\widehat{d \sigma(x)}} \bigr\vert \\ &\quad\leq J_{6}H_{q}(F,\partial\Omega,\beta )+J_{7}C_{q}(F,\partial\Omega) \\ &\quad\leq{J_{8} \Vert {F} \Vert _{\beta}}. \end{aligned}$$

So

11 $$ C_{q}(Q,\partial\Omega)\leq{J_{8} \Vert {F} \Vert _{\beta}}. $$

Next we consider $H_{q}(Q,\partial\Omega,\beta)$.

There is a ball with radius 3*δ*, centered at $y^{1}$ when $y^{1},y^{2}\in{\partial\Omega}$ and $6\delta< d $, $\delta= \vert y^{1}-y^{2} \vert $. Remark that $\partial\Omega_{1}$ is located inside the ball and the rest of *∂*Ω is $\partial\Omega_{2}$.

From equality (9) and (1), we have

$$\begin{aligned}& \bigl\vert {Q \bigl(y^{1} \bigr)-Q \bigl(y^{2} \bigr)} \bigr\vert \\& \quad= \biggl\vert { \frac{1}{2}F \bigl(y^{1} \bigr)- \Phi_{F} \bigl(y^{1} \bigr)- \biggl(\frac {1}{2}F \bigl(y^{2} \bigr)-\Phi_{F} \bigl(y^{2} \bigr) \biggr)} \biggr\vert \\& \quad= \biggl\vert {{ \frac {{2^{n-1}}{(y^{1}_{0})^{n-1}}}{w_{n+1}}}} \biggl[ \int_{\partial\Omega }{E_{1} \bigl(x,y^{1} \bigr)\,d \sigma(x) \bigl(F \bigl(y^{1} \bigr)-F(x) \bigr)} \\& \quad\quad{} + \int_{\partial\Omega }{E_{2} \bigl(x,y^{1} \bigr) \widehat{d\sigma(x)} \bigl(\widehat{F(x)}-F \bigl(y^{1} \bigr) \bigr)} \biggr] \\& \quad\quad{} - {{ \frac {{2^{n-1}}{(y^{2}_{0})^{n-1}}}{w_{n+1}}}} \biggl[ \int_{\partial\Omega }{E_{1} \bigl(x,y^{2} \bigr)\,d \sigma(x) \bigl(F \bigl(y^{2} \bigr)-F(x) \bigr)} \\& \quad\quad{}+ \int_{\partial\Omega }{E_{2} \bigl(x,y^{2} \bigr) \widehat{d\sigma(x)} \bigl(\widehat{F(x)}-F \bigl(y^{2} \bigr) \bigr)} \biggr] \biggr\vert \\& \quad= \biggl\vert {{ \frac {{2^{n-1}}{(y^{1}_{0})^{n-1}}}{w_{n+1}}}} \biggl[ \int_{\partial\Omega _{1}}{E_{1} \bigl(x,y^{1} \bigr)\,d \sigma(x) \bigl(F \bigl(y^{1} \bigr)-F(x) \bigr)} \\& \quad\quad{}+ \int_{\partial \Omega_{1}}{E_{2} \bigl(x,y^{1} \bigr) \widehat{d\sigma(x)} \bigl(\widehat{F(x)}-F \bigl(y^{1} \bigr) \bigr)} \biggr] \\& \quad\quad{} + {{ \frac {{2^{n-1}}{(y^{1}_{0})^{n-1}}}{w_{n+1}}}} \biggl[ \int_{\partial\Omega _{2}}{E_{1} \bigl(x,y^{1} \bigr)\,d \sigma(x) \bigl(F \bigl(y^{1} \bigr)-F(x) \bigr)} \\& \quad\quad{}+ \int_{\partial \Omega_{2}}{E_{2} \bigl(x,y^{1} \bigr) \widehat{d\sigma(x)} \bigl(\widehat{F(x)}-F \bigl(y^{1} \bigr) \bigr)} \biggr] \\& \quad\quad{} - {{ \frac {{2^{n-1}}{(y^{2}_{0})^{n-1}}}{w_{n+1}}}} \biggl[ \int_{\partial\Omega _{1}}{E_{1} \bigl(x,y^{2} \bigr)\,d \sigma(x) \bigl(F \bigl(y^{2} \bigr)-F(x) \bigr)} \\& \quad\quad{}+ \int_{\partial \Omega_{1}}{E_{2} \bigl(x,y^{2} \bigr) \widehat{d\sigma(x)} \bigl(\widehat{F(x)}-F \bigl(y^{2} \bigr) \bigr)} \biggr] \\& \quad\quad{} - {{ \frac {{2^{n-1}}{(y^{2}_{0})^{n-1}}}{w_{n+1}}}} \biggl[ \int_{\partial\Omega _{2}}{E_{1} \bigl(x,y^{2} \bigr)\,d \sigma(x) \bigl(F \bigl(y^{2} \bigr)-F(x) \bigr)} \\& \quad\quad{}+ \int_{\partial \Omega_{2}}{E_{2} \bigl(x,y^{2} \bigr) \widehat{d\sigma(x)} \bigl(\widehat{F(x)}-F \bigl(y^{2} \bigr) \bigr)} \biggr] \biggr\vert \\& \quad\leq\biggl\vert \frac{{2^{n-1}}{(y^{1}_{0})^{n-1}}}{w_{n+1}} \biggr\vert \biggl\vert { \int_{\partial\Omega_{1}}{E_{1} \bigl(x,y^{1} \bigr)\,d\sigma (x) \bigl(F \bigl(y^{1} \bigr)-F(x) \bigr)}} \\& \quad\quad{}+{ \int_{\partial\Omega _{1}}{E_{2} \bigl(x,y^{1} \bigr) \widehat{d\sigma(x)} \bigl(\widehat{F(x)}-F \bigl(y^{1} \bigr) \bigr)}} \biggr\vert \\& \quad\quad{} + \biggl\vert \frac {{2^{n-1}}{(y^{2}_{0})^{n-1}}}{w_{n+1}} \biggr\vert \biggl\vert { \int_{\partial\Omega_{1}}{E_{1} \bigl(x,y^{2} \bigr)\,d\sigma (x) \bigl(F \bigl(y^{2} \bigr)-F(x) \bigr)}} \\& \quad\quad{}+{ \int_{\partial\Omega _{1}}{E_{2} \bigl(x,y^{2} \bigr) \widehat{d\sigma(x)} \bigl(\widehat{F(x)}-F \bigl(y^{2} \bigr) \bigr)}} \biggr\vert \\& \quad\quad{} + \biggl\vert {{ \frac {{2^{n-1}}{(y^{1}_{0})^{n-1}}}{w_{n+1}}}} \biggl[ \int_{\partial\Omega _{2}}{E_{1} \bigl(x,y^{1} \bigr)\,d \sigma(x) \bigl(F \bigl(y^{1} \bigr)-F(x) \bigr)} \\& \quad\quad{}+ \int_{\partial \Omega_{2}}{E_{2} \bigl(x,y^{1} \bigr) \widehat{d\sigma(x)} \bigl(\widehat{F(x)}-F \bigl(y^{1} \bigr) \bigr)} \biggr] \\& \quad\quad{}- {{ \frac{{2^{n-1}}{(y^{2}_{0})^{n-1}}}{w_{n+1}}}} \biggl[ \int_{\partial\Omega_{2}}{E_{1} \bigl(x,y^{2} \bigr)\,d\sigma (x) \bigl(F \bigl(y^{2} \bigr)-F(x) \bigr)} \\& \quad\quad{}+ \int_{\partial\Omega _{2}}{E_{2} \bigl(x,y^{2} \bigr) \widehat{d\sigma(x)} \bigl(\widehat{F(x)}-F \bigl(y^{2} \bigr) \bigr)} \biggr] \biggr\vert \\& \quad=I_{1}+I_{2}+I_{3}; \\& I_{1} \leq{J_{9} \biggl[ \int_{\partial\Omega _{1}} \bigl\vert {E_{1} \bigl(x,y^{1} \bigr)} \bigr\vert \bigl\vert {d\sigma(x)} \bigr\vert \bigl\vert {F \bigl(y^{1} \bigr)-F(x)} \bigr\vert + \int_{\partial \Omega_{1}} \bigl\vert {E_{2} \bigl(x,y^{1} \bigr)} \bigr\vert \bigl\vert {\widehat{d\sigma(x)}} \bigr\vert \bigl\vert {\widehat{F(x)}-F \bigl(y^{1} \bigr)} \bigr\vert \biggr]} \\& \hphantom{I_{1}}\leq{J_{10}} \int^{3\delta}_{0} {\frac {1}{ \vert {x-y^{1}} \vert ^{n} \vert {x-\widehat{y^{1}}} \vert ^{n-1}}}\rho ^{n-1}H_{q}(F,\partial\Omega,\beta) \bigl\vert {y^{1}-x} \bigr\vert ^{\beta}{d\rho} \\& \hphantom{I_{1}}\quad{} +2{J_{10}} \int^{3\delta}_{0} {\frac {1}{ \vert {x-y^{1}} \vert ^{n-1} \vert {x-\widehat{y^{1}}} \vert ^{n}}}\rho ^{n-1}C_{q}(F,\partial\Omega){d\rho} \\& \hphantom{I_{1}} \leq{J_{11}H_{q}(F,\partial\Omega,\beta) \int^{3\delta}_{0} {\frac{1}{ \vert {x-y^{1}} \vert ^{n-\beta}}} \rho^{n-1}\,d\rho}+{J_{12}C_{q}(F,\partial\Omega) \int^{3\delta}_{0} {\frac {1}{ \vert {x-y^{1}} \vert ^{n-1}}} \rho^{n-1}\,d\rho} \\& \hphantom{I_{1}}\leq{J_{11}H_{q}(F,\partial\Omega,\beta) \int^{3\delta}_{0}\rho^{\beta-1}\,d \rho}+{J_{12}C_{q}(F,\partial\Omega) \int^{3\delta}_{0}\,d\rho} \\& \hphantom{I_{1}} \leq{J_{13} \bigl(H_{q}(F,\partial\Omega, \beta)+C_{q}(F,\partial\Omega) \bigr) \bigl\vert {y^{1}-y^{2}} \bigr\vert ^{\beta}} \\& \hphantom{I_{1}} \leq{J_{13} \Vert {F} \Vert _{\beta } \bigl\vert {y^{1}-y^{2}} \bigr\vert ^{\beta}}, \end{aligned}$$

that is,

12 $$ I_{1}\leq{J_{13} \Vert {F} \Vert _{\beta} \bigl\vert {y^{1}-y^{2}} \bigr\vert ^{\beta}}. $$

In a similar way, we have

13 $$\begin{aligned}& I_{2}\leq{J_{14} \Vert {F} \Vert _{\beta} \bigl\vert {y^{1}-y^{2}} \bigr\vert ^{\beta}} , \\& I_{3} = \biggl\vert {{ \frac{{2^{n-1}}{(y^{1}_{0})^{n-1}}}{w_{n+1}}}} \int_{\partial\Omega_{2}} \bigl[E_{1} \bigl(x,y^{1} \bigr)-E_{1} \bigl(x,y^{2} \bigr) \bigr]\,d\sigma(x) \bigl(F \bigl(y^{1} \bigr)-F(x) \bigr) \\& \hphantom{I_{3}} \quad{} + {{ \frac {{2^{n-1}}{(y^{1}_{0})^{n-1}}}{w_{n+1}}}} \int_{\partial\Omega _{2}}E_{1} \bigl(x,y^{2} \bigr)\,d \sigma(x) \bigl(F \bigl(y^{1} \bigr)-F(x) \bigr) \\& \hphantom{I_{3}} \quad{}+ {{ \frac {{2^{n-1}}{(y^{1}_{0})^{n-1}}}{w_{n+1}}}} \int_{\partial\Omega _{2}} \bigl[E_{2} \bigl(x,y^{1} \bigr)-E_{2} \bigl(x,y^{2} \bigr) \bigr]\widehat{d\sigma(x)} \bigl(\widehat{F(x)}-F \bigl(y^{1} \bigr) \bigr) \\& \hphantom{I_{3}} \quad{}+ {{ \frac {{2^{n-1}}{(y^{1}_{0})^{n-1}}}{w_{n+1}}}} \int_{\partial\Omega _{2}}E_{2} \bigl(x,y^{2} \bigr) \widehat{d \sigma(x)} \bigl(\widehat{F(x)}-F \bigl(y^{1} \bigr) \bigr) \\& \hphantom{I_{3}} \quad{}- {{ \frac {{2^{n-1}}{(y^{2}_{0})^{n-1}}}{w_{n+1}}}} \int_{\partial\Omega _{2}}E_{1} \bigl(x,y^{2} \bigr)\,d \sigma(x) \bigl(F \bigl(y^{2} \bigr)-F(x) \bigr) \\& \hphantom{I_{3}} \quad{}- {{ \frac {{2^{n-1}}{(y^{2}_{0})^{n-1}}}{w_{n+1}}}} \int_{\partial\Omega _{2}}E_{2} \bigl(x,y^{2} \bigr) \widehat{d \sigma(x)} \bigl(\widehat{F(x)}-F \bigl(y^{2} \bigr) \bigr) \biggr\vert \\& \hphantom{I_{3}} \leq{J_{15}} \biggl\vert \int_{\partial\Omega _{2}} \bigl[E_{1} \bigl(x,y^{1} \bigr)-E_{1} \bigl(x,y^{2} \bigr) \bigr]\,d\sigma(x) \bigl(F \bigl(y^{1} \bigr)-F(x) \bigr) \biggr\vert \\& \hphantom{I_{3}} \quad{}+{J_{15}} \biggl\vert \int_{\partial\Omega _{2}} \bigl[E_{2} \bigl(x,y^{1} \bigr)-E_{2} \bigl(x,y^{2} \bigr) \bigr]\widehat{d\sigma(x)} \bigl(\widehat{F(x)}-F \bigl(y^{1} \bigr) \bigr) \biggr\vert \\& \hphantom{I_{3}} \quad{}+ \biggl\vert {{ \frac {{2^{n-1}}{(y^{1}_{0})^{n-1}}}{w_{n+1}}}} \int_{\partial\Omega _{2}}E_{1} \bigl(x,y^{2} \bigr)\,d \sigma(x) \bigl(F \bigl(y^{1} \bigr)-F(x) \bigr) \\& \hphantom{I_{3}} \quad{}-{ {\frac {{2^{n-1}}{(y^{2}_{0})^{n-1}}}{w_{n+1}}}} \int_{\partial\Omega _{2}}E_{1} \bigl(x,y^{2} \bigr)\,d \sigma(x) \bigl(F \bigl(y^{2} \bigr)-F(x) \bigr) \biggr\vert \\& \hphantom{I_{3}} \quad{}+ \biggl\vert {{ \frac {{2^{n-1}}{(y^{1}_{0})^{n-1}}}{w_{n+1}}}} \int_{\partial\Omega _{2}}E_{2} \bigl(x,y^{2} \bigr) \widehat{d \sigma(x)} \bigl(\widehat{F(x)}-F \bigl(y^{1} \bigr) \bigr) \\& \hphantom{I_{3}} \quad{}-{ {\frac {{2^{n-1}}{(y^{2}_{0})^{n-1}}}{w_{n+1}}}} \int_{\partial\Omega _{2}}E_{2} \bigl(x,y^{2} \bigr) \widehat{d \sigma(x)} \bigl(\widehat{F(x)}-F \bigl(y^{2} \bigr) \bigr) \biggr\vert \\& \hphantom{I_{3}} ={J_{15}}(I_{4}+I_{5})+I_{6}+I_{7}, \end{aligned}$$

that is,

14 $$ I_{3}\leq{J_{15}}(I_{4}+I_{5})+I_{6}+I_{7} . $$

Because $x\in{\partial\Omega_{2}\setminus\lambda_{3\delta}}$, and $y^{1},y^{2}\in\partial\Omega_{1}$, $\vert \frac {x-y^{2}}{x-y^{1}} \vert ^{l+1}$ and $\vert \frac {x-y^{1}}{x-y^{2}} \vert ^{l+1}$ ($l=0,1,\ldots,n$) are continuous on $\partial\Omega_{2}$, there is a positive constant $J_{16} $, such that

15 $$ { \biggl\vert \frac{x-y^{2}}{x-y^{1}} \biggr\vert ^{l+1}}\leq {J_{16}}, \quad\quad \biggl\vert \frac{x-y^{1}}{x-y^{2}} \biggr\vert ^{l+1}\leq{J_{16}} \quad(l=0,1,\ldots,n). $$

From inequality (1), the Hile lemma and inequality (15), we get

$$\begin{aligned} I_{4}&= \biggl\vert \int_{\partial\Omega _{2}} \bigl[E_{1} \bigl(x,y^{1} \bigr)-E_{1} \bigl(x,y^{2} \bigr) \bigr]\,d\sigma(x) \bigl(F \bigl(y^{1} \bigr)-F(x) \bigr) \biggr\vert \\ &= \biggl\vert \int_{\partial\Omega_{2}} \biggl( {\frac {x-y^{1}}{ \vert {x-y^{1}} \vert ^{n+1} \vert {x-\widehat {y^{1}}} \vert ^{n-1}}-\frac {x-y^{2}}{ \vert {x-y^{2}} \vert ^{n+1} \vert {x-\widehat {y^{2}}} \vert ^{n-1}}} \biggr)\,d\sigma(x) \bigl(F \bigl(y^{1} \bigr)-F(x) \bigr) \biggr\vert \\ &\leq{J_{17}} \int_{\partial\Omega_{2}} \biggl\vert \frac {x-y^{1}}{ \vert {x-y^{1}} \vert ^{n+1} \vert {x-\widehat {y^{1}}} \vert ^{n-1}}-\frac {x-y^{2}}{ \vert {x-y^{2}} \vert ^{n+1} \vert {x-\widehat {y^{2}}} \vert ^{n-1}} \biggr\vert \bigl\vert {d\sigma(x)} \bigr\vert \bigl\vert {F \bigl(y^{1} \bigr)-F(x)} \bigr\vert \\ &\leq{J_{17}} \int_{\partial\Omega_{2}} {\frac{1}{ \vert {x-\widehat {y^{1}}} \vert ^{n-1}} \biggl\vert \frac{x-y^{1}}{ \vert {x-y^{1}} \vert ^{n+1}}- \frac {x-y^{2}}{ \vert {x-y^{2}} \vert ^{n+1}} \biggr\vert } \bigl\vert {d\sigma(x)} \bigr\vert \bigl\vert {F \bigl(y^{1} \bigr)-F(x)} \bigr\vert \\ &\quad{} +{J_{17}} \int_{\partial\Omega_{2}} {\frac {1}{ \vert {x-y^{2}} \vert ^{n+1}} \biggl\vert \frac{x-y^{2}}{ \vert {x-\widehat {y^{1}}} \vert ^{n-1}}- \frac{x-y^{2}}{ \vert {x-\widehat{y^{2}}} \vert ^{n-1}} \biggr\vert } \bigl\vert {d\sigma(x)} \bigr\vert \bigl\vert {F \bigl(y^{1} \bigr)-F(x)} \bigr\vert \\ &\leq \int_{\partial\Omega_{2}} \Biggl(J_{18}\sum _{l=0}^{n-1} \biggl\vert \frac{x-y^{2}}{x-y^{1}} \biggr\vert ^{l+1}\frac{ \vert {y^{1}-y^{2}} \vert }{ \vert {x-y^{2}} \vert ^{n+1}} +J_{19}\frac{ \vert {y^{1}-y^{2}} \vert }{ \vert {x-y^{2}} \vert ^{n}} \Biggr) \bigl\vert {d\sigma(x)} \bigr\vert \bigl\vert {F \bigl(y^{1} \bigr)-F(x)} \bigr\vert \\ &\leq{J_{20}}H_{q}(F,\partial\Omega,\beta) \int_{\partial\Omega _{2}} \biggl( {\frac{1}{ \vert {x-y^{2}} \vert ^{n+1}}+\frac {1}{ \vert {x-y^{2}} \vert ^{n}}} \biggr) \bigl\vert {d\sigma(x)} \bigr\vert \bigl\vert {y^{1}-x} \bigr\vert ^{\beta } \bigl\vert {y^{1}-y^{2}} \bigr\vert \\ &\leq H_{q}(Q,\partial\Omega,\beta) \biggl(J_{21} \int^{L}_{3\delta}\rho^{\beta-2}\,d \rho+J_{22} \int^{L}_{3\delta}\rho^{\beta-1}\,d\rho\biggr) \bigl\vert {y^{1}-y^{2}} \bigr\vert \\ &\leq{J_{23}}H_{q}(F,\partial\Omega,\beta) \bigl\vert {y^{1}-y^{2}} \bigr\vert ^{\beta} \\ &\leq{J_{24}} \Vert {F} \Vert _{\beta} \bigl\vert {y^{1}-y^{2}} \bigr\vert ^{\beta}, \end{aligned}$$

that is,

16 $$ I_{4}\leq{J_{24}} \Vert {F} \Vert _{\beta } \bigl\vert {y^{1}-y^{2}} \bigr\vert ^{\beta}. $$

In a similar way, we have

17 $$\begin{aligned}& I_{5}\leq{J_{25}} \Vert {F} \Vert _{\beta } \bigl\vert {y^{1}-y^{2}} \bigr\vert ^{\beta}, \\& I_{6}\leq\biggl\vert {\frac {2^{n-1}[(y^{2}_{0})^{n-1}-(y^{1}_{0})^{n-1}]}{w_{n+1}}} \int_{\partial \Omega_{2}} {\frac{x-y^{2}}{ \vert {x-y^{2}} \vert ^{n+1} \vert {x-\widehat {y^{2}}} \vert ^{n-1}}}\,d\sigma(x)F(x) \biggr\vert \\& \hphantom{I_{6}}\quad{} + \biggl\vert {\frac {{2^{n-1}}{(y^{1}_{0})^{n-1}}}{w_{n+1}}} \int_{\partial \Omega_{2}} {\frac{x-y^{2}}{ \vert {x-y^{2}} \vert ^{n+1} \vert {x-\widehat {y^{2}}} \vert ^{n-1}}}\,d\sigma(x) \bigl(F \bigl(y^{1} \bigr)-F \bigl(y^{2} \bigr) \bigr) \biggr\vert \\& \hphantom{I_{6}}\quad{}+ \biggl\vert {\frac {2^{n-1}[(y^{1}_{0})^{n-1}-(y^{2}_{0})^{n-1}]}{w_{n+1}}} \int_{\partial \Omega_{2}} {\frac{x-y^{2}}{ \vert {x-y^{2}} \vert ^{n+1} \vert {x-\widehat {y^{2}}} \vert ^{n-1}}}\,d\sigma(x)F \bigl(y^{2} \bigr) \biggr\vert . \end{aligned}$$

Because $\lim_{\delta\rightarrow0} {\frac {x-y^{2}}{ \vert {x-y^{2}} \vert ^{n+1} \vert {x-\widehat {y^{2}}} \vert ^{n-1}}}$ exists, there is a constant $\delta_{2}>0$, when $0<\delta<\delta_{2}$, such that

$$\biggl\vert \int_{\partial\Omega_{2}}\frac {(x-y^{2})}{ \vert {x-y^{2}} \vert ^{n+1} \vert {x-\widehat {y^{2}}} \vert ^{n-1}}\,d\sigma(x) \biggr\vert \leq{J_{26}}. $$

Hence

$$\begin{aligned} I_{6}&\leq{J_{27}} \bigl[2C_{q}(F,\partial\Omega ) \bigl\vert \bigl(y^{1}_{0} \bigr)^{n-1}- \bigl(y^{2}_{0} \bigr)^{n-1} \bigr\vert + \bigl\vert {F \bigl(y^{1} \bigr)-F \bigl(y^{2} \bigr)} \bigr\vert \bigr] \\ &\leq{J_{28}} \bigl[C_{q}(F,\partial\Omega)+H_{q}(F, \partial\Omega,\beta) \bigr] \bigl\vert {y^{1}-y^{2}} \bigr\vert ^{\beta} \\ &={J_{28}} \Vert {F} \Vert _{\beta} \bigl\vert {y^{1}-y^{2}} \bigr\vert ^{\beta}, \end{aligned}$$

that is,

18 $$ I_{6}\leq{J_{28}} \Vert {F} \Vert _{\beta } \bigl\vert {y^{1}-y^{2}} \bigr\vert ^{\beta}. $$

In a similar way, we have

19 $$ I_{7}\leq{J_{29}} \Vert {F} \Vert _{\beta } \bigl\vert {y^{1}-y^{2}} \bigr\vert ^{\beta}. $$

From inequalities (14), (16), (17), (18) and (19), we have

20 $$ \vert I_{3} \vert \leq\bigl[J_{15}(J_{24}+J_{25})+J_{28}+J_{29} \bigr] \Vert {F} \Vert _{\beta} \bigl\vert y^{1}-y^{2} \bigr\vert ^{\beta } . $$

From inequalities (12), (13) and (20), we have

$${\frac{ \vert {Q(y^{1})-Q(y^{2})} \vert }{ \vert {y^{1}-y^{2}} \vert ^{\beta}}}\leq\bigl[J_{13}+J_{14}+J_{15}(J_{24}+J_{25})+J_{28}+J_{29} \bigr] \Vert {F} \Vert _{\beta} ={J_{30}} \Vert {F} \Vert _{\beta}. $$

So

21 $$ H_{q} \bigl(Q(y),\partial\Omega,\beta\bigr) \leq{J_{30}} \Vert {F} \Vert _{\beta}. $$

From inequalities (11) and (21), we have $\Vert {Q(y)} \Vert _{\beta}\leq(J_{8}+J_{30}) \Vert {F} \Vert _{\beta}=J_{31} \Vert {F} \Vert _{\beta}$. □

### Remark 3.1

If $y\in\partial\Omega$, $F\in{H_{q}(\beta,\partial\Omega ,\mathit {Cl}_{n+1,0}(\mathbb{R})})$, then

$$\bigl\Vert \Phi_{F}(y) \bigr\Vert _{\beta} \leq{J_{32}} \Vert {F} \Vert _{\beta}. $$

### Remark 3.2

If $y\in\partial\Omega$, $F\in{H_{q}(\beta,\partial\Omega ,\mathit {Cl}_{n+1,0}(\mathbb{R})})$, then

22 $$ \textstyle\begin{cases} \Vert {\Psi^{+}_{F}(y)} \Vert _{\beta}\leq{J_{33}} \Vert {F} \Vert _{\beta}, \\ \Vert {\Psi^{-}_{F}(y)} \Vert _{\beta}\leq{J_{33}} \Vert {F} \Vert _{\beta}. \end{cases} $$

## The existence of the solution to the nonlinear boundary value problem for the hypergenic function vector

Let $A(y),B(y),G(y)\in{H_{q}(\beta,\partial\Omega, \mathit {Cl}_{n+1,0}(\mathbb {R})})$ be Hölder continuous function vectors on *∂*Ω, we find a function vector $\Psi_{F}^{*}(y)$, which is hypergenic on $\Omega^{+} \cup\Omega^{-}$, and continuous on $\Omega^{+}\cup \partial\Omega$ and $\Omega^{-}\cup\partial\Omega$, satisfying $\Psi_{F}^{*}(\infty)=0$, and the nonlinear boundary value condition:

23 $$ A(y)\otimes{\Psi^{*+}_{F}}(y)+B(y)\otimes{\Psi ^{*-}_{F}}(y)=G(y)\otimes P \bigl({\Psi^{*+}_{F}}(y),{ \Psi^{*-}_{F}}(y) \bigr), $$

where $P(\Psi^{*+}_{F}(y),\Psi^{*-}_{F}(y))$ is a Hölder continuous function vector on *∂*Ω which is related to $\Psi^{*+}_{F}(y)$, $\Psi^{*-}_{F}(y)$.

The above problem is called the nonlinear boundary value problem *SR*. If $P=1$, then the above problem is called the linear boundary value problem *SR*.

By Theorem 3.1, $\Psi_{F}(y)$ is hypergenic on $\Omega^{+} \cup \Omega^{-}$, and $\Psi_{F}(y)$ is continuous on $\Omega^{+}\cup \partial\Omega$ and $\Omega^{-}\cup\partial\Omega$, and $\Psi_{ F}(\infty)=0$. If $P(\Psi^{+}_{F}(y),\Psi^{-}_{F}(y))$ satisfies equality (23) under certain conditions, then $\Psi_{F}(y)$ is a solution to the nonlinear boundary value problem *SR*.

Putting (10) into (23), we have

24 $$ A(y)\otimes\biggl(\Phi_{F}(y)+\frac{1}{2}F(y) \biggr)+B(y) \otimes\biggl(\Phi_{F}(y)-\frac{1}{2}F(y) \biggr)=G(y)\otimes P \bigl(\Psi^{+}_{F}(y),\Psi^{-}_{F}(y) \bigr). $$

Let

25 $$ \begin{aligned}[b] NF(y)& = \bigl(A(y)+B(y) \bigr)\otimes\biggl(- \frac{1}{2}F(y)+\Phi_{F}(y) \biggr)+ \bigl(1+A(y) \bigr)\otimes F(y) \\ &\quad{} -G(y)\otimes P \bigl(\Psi^{+}_{F}(y), \Psi^{-}_{F}(y) \bigr), \end{aligned} $$

and equality (23) is transformed into the following singular integral equation:

26 $$ NF=F . $$

### Theorem 4.1

*If* $A(y),B(y),G(y)\in{H_{q}(\beta,\partial\Omega, \mathit {Cl}_{n+1,0}(\mathbb {R})})$, *for any* $y^{1},y^{2}\in\partial\Omega$, $P(\Psi^{+}_{F}(y), \Psi^{-}_{F}(y))$ *satisfies*

27 $$ \begin{gathered}[b] \bigl\vert {P} \bigl(\Psi^{+}_{F} \bigl(y^{1} \bigr),\Psi^{-}_{F} \bigl(y^{1} \bigr) \bigr)-P \bigl(\Psi^{+}_{F} \bigl(y^{2} \bigr),\Psi^{-}_{F} \bigl(y^{2} \bigr) \bigr) \bigr\vert \\ \quad\leq J_{34} \bigl\vert \Psi^{+}_{F} \bigl(y^{1} \bigr)-\Psi^{+}_{F} \bigl(y^{2} \bigr) \bigr\vert +J_{35} \bigl\vert \Psi^{-}_{F} \bigl(y^{1} \bigr)- \Psi^{-}_{F} \bigl(y^{2} \bigr) \bigr\vert , \end{gathered} $$

*where* $J_{34}$ *and* $J_{35}$ *are positive constants independent of* $y^{i}$ ($i=1,2$) *and F*. *If* $P(0,0)=0$, $0<\gamma=J_{36}( \Vert {A+B} \Vert _{\beta}+ \Vert 1+A \Vert _{\beta})<1$, $\Vert {G(y)} \Vert _{\beta}<\delta$, *when* $0<\delta\leq{\frac{1-\gamma }{J_{3}J_{41}}}$, *Problem* *SR* *has at least one solution and the integral expression of the solution is* (8).

### Proof

Let $T=\{F\mid { \Vert F \Vert }_{\beta}\leq M_{4}$ and *F* is uniformly Hölder continuous on *∂*Ω, that is, to say, there is a positive constant ${M_{2}}$, for any ${x_{1},x_{2}\in\partial\Omega}$, ${F}\in{H_{q}(\beta,\partial\Omega, \mathit {Cl}_{n+1,0}(\mathbb{R})})$, we have $\vert F(x_{1})-F(x_{2}) \vert \leq{M_{2} \vert x_{1}-x_{2} \vert ^{\beta}}\}$. Obviously *T* is a convex subset of the continuous function vector space $C_{q}(\partial\Omega)$.

1. We prove that *N* maps the set *T* to itself.

From inequality (7), Theorem 3.1 and Remark 3.2, it follows that

$$\begin{aligned}& \Vert NF \Vert \\& \quad\leq J_{3} \bigl\Vert A(y)+B(y) \bigr\Vert _{\beta} \biggl\Vert {-\frac {1}{2}}F(y)+\Phi_{F}(y) \biggr\Vert _{\beta}+J_{3} \bigl\Vert 1+A(y) \bigr\Vert _{\beta} \Vert F \Vert _{\beta}+J_{3} \Vert G \Vert _{\beta} \Vert P \Vert _{\beta} \\& \quad\leq J_{3} \bigl\Vert A(y)+B(y) \bigr\Vert _{\beta}J_{31} \Vert F \Vert _{\beta}+J_{3} \bigl\Vert 1+A(y) \bigr\Vert _{\beta} \Vert F \Vert _{\beta}+J_{3} \Vert G \Vert _{\beta} \Vert P \Vert _{\beta} \\& \quad\leq J_{36} \bigl( \Vert {A+B} \Vert _{\beta}+ \Vert 1+A \Vert _{\beta} \bigr) \Vert F \Vert _{\beta}+J_{3} \Vert G \Vert _{\beta} \Vert P \Vert _{\beta} \\& \quad\leq\gamma \Vert F \Vert _{\beta}+J_{3}\delta \Vert P \Vert _{\beta}. \end{aligned}$$

By inequality (27) and Remark 3.2, we have

$$\begin{aligned}& \bigl\vert P \bigl(\Psi^{+}_{F}(y),\Psi^{-}_{F}(y) \bigr) \bigr\vert \\& \quad= \bigl\vert P \bigl(\Psi^{+}_{F}(y), \Psi^{-}_{F}(y) \bigr)-P(0,0) \bigr\vert \\& \quad\leq J_{34} \bigl\vert \Psi^{+}_{F}(y) \bigr\vert +J_{35} \bigl\vert \Psi^{-}_{F}(y) \bigr\vert \\& \quad\leq J_{34}J_{33} \Vert F \Vert _{\beta}+J_{35}J_{33} \Vert F \Vert _{\beta} \\& \quad=J_{37} \Vert F \Vert _{\beta}. \end{aligned}$$

So

28 $$ C_{q}(P,\partial\Omega,\beta)=\max_{y\in{\partial\Omega}} \vert P \vert \leq J_{37} \Vert F \Vert _{\beta}. $$

By inequality (27) and Remark 3.2, we have

29 $$\begin{aligned}& \bigl\vert {P} \bigl(\Psi^{+}_{F} \bigl(y^{1} \bigr),\Psi^{-}_{F} \bigl(y^{1} \bigr) \bigr)-P \bigl(\Psi^{+}_{F} \bigl(y^{2} \bigr),\Psi^{-}_{F} \bigl(y^{2} \bigr) \bigr) \bigr\vert \\& \quad\leq J_{34} \bigl\vert \Psi^{+}_{F} \bigl(y^{1} \bigr)-\Psi^{+}_{F} \bigl(y^{2} \bigr) \bigr\vert +J_{35} \bigl\vert \Psi^{-}_{F} \bigl(y^{1} \bigr)- \Psi^{-}_{F} \bigl(y^{2} \bigr) \bigr\vert \\& \quad\leq J_{34}H_{q} \bigl(\Psi^{+}_{F}(y), \partial\Omega,\beta\bigr) \bigl\vert y^{1}-y^{2} \bigr\vert ^{\beta}+J_{35}H_{q} \bigl( \Psi^{+}_{F}(y),\partial\Omega,\beta\bigr) \bigl\vert y^{1}-y^{2} \bigr\vert ^{\beta} \\& \quad\leq\bigl[J_{34} \bigl\Vert \Psi^{+}_{F}(y) \bigr\Vert _{\beta }+J_{35} \bigl\Vert \Psi^{+}_{F}(y) \bigr\Vert _{\beta} \bigr] \bigl\vert y^{1}-y^{2} \bigr\vert ^{\beta} \\& \quad\leq\bigl(J_{38} \Vert F \Vert _{\beta}+J_{39} \Vert F \Vert _{\beta} \bigr) \bigl\vert y^{1}-y^{2} \bigr\vert ^{\beta} \\& \quad\leq J_{40} \Vert F \Vert _{\beta} \bigl\vert y^{1}-y^{2} \bigr\vert ^{\beta}, \end{aligned}$$

then

$$H_{q}(P,\partial\Omega,\beta)\leq J_{40} \Vert F \Vert _{\beta}. $$

So

30 $$ \begin{aligned}[b] \Vert P \Vert _{\beta}&=C_{q}(P, \partial\Omega,\beta)+H_{q}(P,\partial\Omega,\beta) \\ &\leq J_{37} \Vert F \Vert _{\beta}+J_{40} \Vert F \Vert _{\beta} \\ &\leq J_{41} \Vert F \Vert _{\beta}. \end{aligned} $$

As $\gamma=J_{35}( \Vert {A+B} \Vert _{\beta}+ \Vert 1+A \Vert _{\beta})<1$,

31 $$ \begin{aligned}[b] \Vert NF \Vert _{\beta}&\leq\gamma \Vert F \Vert _{\beta}+J_{3}\delta \Vert P \Vert _{\beta} \\ &\leq\gamma M_{4}+J_{3} {\frac{1-\gamma}{J_{3}J_{41}}}J_{41}M_{4}=M_{4}. \end{aligned} $$

If *F* is uniformly Hölder continuous on *∂*Ω, then $\Phi_{F}(y)$, $\Psi^{+}_{F}$, $\Psi^{-}_{F}$ are uniformly Hölder continuous on *∂*Ω. So *NF* is uniformly Hölder continuous on *∂*Ω.

Hence *N* maps the set *T* to itself.

(2) We prove that *N* is a continuous mapping.

Any $F_{n}\in{T}$, $\{F_{n}\}$ uniformly converges to *F* on *∂*Ω. As for $\varepsilon>0$, when *n* is fully large and $\vert {F_{n}-F} \vert $ is sufficiently small. There is a ball with radius 3*δ*, centered at *y* when $6\delta \langle d,\delta\rangle0$, and remark that $\partial\Omega_{1}$ is located inside the ball and the rest of *∂*Ω is $\partial\Omega_{2}$

By inequality (27), Theorem 3.3, we have

$$\begin{aligned}& \bigl\vert P \bigl(\Psi^{+}_{F_{n}}(y), \Psi^{-}_{F_{n}}(y) \bigr)-P \bigl(\Psi^{+}_{F}(y), \Psi^{-}_{F}(y) \bigr) \bigr\vert \\& \quad\leq{J_{34}} \bigl\vert \Psi^{+}_{F_{n}}(y)- \Psi^{+}_{F}(y) \bigr\vert +{J_{35}} \bigl\vert \Psi^{-}_{F_{n}}(y)-\Psi^{-}_{F}(y) \bigr\vert \\& \quad=J_{34} \biggl\vert \Phi_{F_{n}}(y)- \Phi_{F}(y)+ \frac {1}{2} \bigl(F_{n}(y)-F(y) \bigr) \biggr\vert +J_{35} \biggl\vert \Phi_{F_{n}}(y)- \Phi_{F}(y)+ \frac {1}{2} \bigl(F(y)-F_{n}(y) \bigr) \biggr\vert \\& \quad\leq(J_{34}+J_{35}) \bigl\vert \Phi_{F_{n}}(y)-\Phi_{F}(y) \bigr\vert +(J_{34}+J_{35}) \biggl\vert \frac{1}{2} \bigl(F_{n}(y)-F(y) \bigr) \biggr\vert \\& \quad\leq(J_{34}+J_{35}) \biggl\vert { \frac {{2^{n-1}}{y_{0}^{n-1}}}{w_{n+1}}} \int_{\partial\Omega }E_{1}(x,y)\,d\sigma(x) \bigl[ \bigl(F_{n}(x)-F_{n}(y) \bigr)+ \bigl(F(y)-F(x) \bigr) \bigr] \biggr\vert \\& \quad\quad{} +(J_{34}+J_{35}) \biggl\vert \frac {{2^{n-1}}{y_{0}^{n-1}}}{w_{n+1}} \int_{\partial\Omega }E_{2}(x,y)\widehat{d\sigma(x)} \bigl[ \bigl( \widehat{F(x)}-\widehat{F(y)} \bigr)+ \bigl(\widehat{F_{n}(y)}- \widehat{F_{n}(x)} \bigr) \bigr] \biggr\vert \\& \quad\quad{} +(J_{34}+J_{35}) \vert F_{n}(y)-F(y) \vert \\& \quad\leq(J_{34}+J_{35}) \bigl(I_{8}+I_{9}+ \Vert F_{n}-F \Vert _{\beta} \bigr), \end{aligned}$$

that is,

32 $$\begin{aligned}& \bigl\vert P \bigl(\Psi^{+}_{F_{n}}(y), \Psi^{-}_{F_{n}}(y) \bigr)-P \bigl(\Psi^{+}_{F}(y), \Psi^{-}_{F}(y) \bigr) \bigr\vert \leq(J_{34}+J_{35}) \bigl(I_{8}+I_{9}+ \Vert F_{n}-F \Vert _{\beta} \bigr), \\& I_{8} \leq\biggl\vert \frac{{2^{n-1}}{y_{0}^{n-1}}}{w_{n+1}} \int_{\partial\Omega}E_{1}(x,y)\,d\sigma(x) \bigl[ \bigl(F_{n}(x)-F_{n}(y) \bigr)+ \bigl(F(y)-F(x) \bigr) \bigr] \biggr\vert \\& \hphantom{I_{8}}\leq\biggl\vert \frac{{2^{n-1}}{y_{0}^{n-1}}}{w_{n+1}} \int_{\partial \Omega_{1}}E_{1}(x,y)\,d\sigma(x) \bigl[ \bigl(F_{n}(x)-F_{n}(y) \bigr)+ \bigl(F(y)-F(x) \bigr) \bigr] \biggr\vert \\& \hphantom{I_{8}}\quad{} + \biggl\vert \frac{{2^{n-1}}{y_{0}^{n-1}}}{w_{n+1}} \int_{\partial\Omega _{2}}E_{1}(x,y)\,d\sigma(x) \bigl[ \bigl(F_{n}(x)-F_{n}(y) \bigr)+ \bigl(F(y)-F(x) \bigr) \bigr] \biggr\vert \\& \hphantom{I_{8}}=I_{10}+I_{11}; \\& I_{10} \leq{J_{42}} \int_{\partial\Omega _{1}} \bigl\vert {E_{1}(x,y)} \bigr\vert \bigl\vert {d\sigma(x)} \bigr\vert \vert {x-y} \vert ^{\beta} \\& \hphantom{I_{10}}\leq{J_{43}} \int_{\partial\Omega_{1}} \frac {1}{ \vert {x-y} \vert ^{n}} \vert {x-y} \vert ^{\beta} \bigl\vert {d\sigma(x)} \bigr\vert \\& \hphantom{I_{10}}\leq{J_{44}} \int^{3\delta}_{0}\rho^{\beta-1}\,d\rho \\& \hphantom{I_{10}}=J_{45}\delta^{\beta}, \end{aligned}$$

that is,

33 $$\begin{aligned}& I_{10}\leq{J_{45}}\delta^{\beta}, \\& I_{11} = \biggl\vert \frac{{2^{n-1}}{y_{0}^{n-1}}}{w_{n+1}} \int_{\partial \Omega_{2}}E_{1}(x,y)\,d\sigma(x) \bigl[ \bigl(F_{n}(x)-F(x) \bigr)- \bigl(F_{n}(y)-F(y) \bigr) \bigr] \biggr\vert \\& \hphantom{I_{11}} \leq{J_{46}} \biggl\vert \int_{\partial\Omega_{2}}E_{1}(x,y)\,d\sigma(x) \biggr\vert \bigl[ \bigl\vert F_{n}(x)-F(x) \bigr\vert + \bigl\vert F_{n}(y)-F(y) \bigr\vert \bigr] \\& \hphantom{I_{11}}\leq{J_{47}} \Vert F_{n}-F \Vert _{\beta} \biggl\vert \int_{\partial\Omega_{2}}E_{1}(x,y)\,d\sigma(x) \biggr\vert \\& \hphantom{I_{11}} \leq J_{48} \Vert F_{n}-F \Vert _{\beta}, \end{aligned}$$

that is,

34 $$ I_{11}\leq{J_{48}} \Vert F_{n}-F \Vert _{\beta}. $$

From inequality (33) and (34), we have

35 $$ I_{8}\leq{J_{45}}\delta^{\beta}+J_{48} \Vert F_{n}-F \Vert _{\beta}. $$

In a similar way, we get

36 $$ I_{9}\leq{J_{49}}\delta^{\beta}+J_{50} \Vert F_{n}-F \Vert _{\beta}. $$

From inequality (32), (35) and (36), we get

$$\begin{aligned} & \bigl\vert P \bigl(\Psi^{+}_{F_{n}}(y), \Psi^{-}_{F_{n}}(y) \bigr)-P \bigl(\Psi^{+}_{F}(y), \Psi^{-}_{F}(y) \bigr) \bigr\vert \\ &\quad\leq(J_{34}+J_{35}) \bigl[(J_{45}+J_{49}) \delta^{\beta }+(J_{48}+J_{50}) \Vert F_{n}-F \Vert _{\beta} \bigr] \\ &\quad=J_{51}\delta^{\beta}+J_{52} \Vert F_{n}-F \Vert _{\beta}. \end{aligned}$$

Select a sufficiently small positive number *δ* such that $J_{51}\delta^{\beta}< {\frac{\varepsilon}{2}}$; and let *n* be large enough such that $J_{52} \Vert F_{n}-F \Vert _{\beta}< {\frac{\varepsilon}{2}}$. So for any ${y}\in\partial\Omega$, we have $\vert P(\Psi^{+}_{F_{n}}(y),\Psi^{-}_{F_{n}}(y))-P(\Psi ^{+}_{F}(y),\Psi^{-}_{F}(y)) \vert <\varepsilon$, thus $\vert {NF_{n}(y)-NF(y)} \vert <\varepsilon$, then *N* is a continuous mapping which maps *T* to itself.

From the Arzela–Ascoli theorem we conclude that *T* is a compact set in $\mathbf{C_{q}}(\partial\Omega)$. As the continuous mapping *N* maps the closed convex set *T* to itself, $N(T)$ is compact in $\mathbf {C_{q}}(\partial\Omega)$. From the Schauder fixed point principle it follows that there is at least $F\in{H_{q}(\beta,\partial\Omega ,\mathit {Cl}_{n+1,0}(\mathbb{R}))}$ that satisfies (26). Hence the nonlinear boundary value problem *SR* has at least one solution $\Psi_{F}(y)$, and the expression of the solution is (8). □

## The existence and uniqueness of the solution to the linear boundary value problem for the hypergenic function vector

### Theorem 5.1

*If* $A(y),B(y),G(y)\in{H_{q}(\beta,\partial\Omega, \mathit {Cl}_{n+1,0}(\mathbb {R})})$, *when* $0<\gamma=J_{3}(J_{32}+ \frac{1}{2}) \Vert {A+B} \Vert _{\beta}+J_{3} \Vert {1+A} \Vert _{\beta}<1$, *the linear boundary value problem* *SR* *has a unique solution*.

### Proof

Let *T* be as in Theorem 4.1. *N* is a continuous mapping which maps *T* to itself from Theorem 4.1.

From inequalities (7), (25) and Remark 3.1, we get

$$\begin{aligned}& \Vert {N{F_{1}}-N{F_{2}}} \Vert _{\beta} \\& \quad\leq{J_{3}} \Vert {A+B} \Vert _{\beta} \biggl\Vert \frac {1}{2}(F_{2}-F_{1})+\Phi_{F_{1}}- \Phi_{F_{2}} \biggr\Vert _{\beta} +J_{3} \Vert {1+A} \Vert _{\beta} \Vert {F_{1}-F_{2}} \Vert _{\beta} \\& \quad\leq{J_{3}} \Vert {A+B} \Vert _{\beta} \biggl[ \biggl\Vert \frac {1}{2}(F_{1}-F_{2}) \biggr\Vert _{\beta}+ \Vert \Phi_{F_{1}}-\Phi_{F_{2}} \Vert _{\beta} \biggr] +J_{3} \Vert {1+A} \Vert _{\beta} \Vert {F_{1}-F_{2}} \Vert _{\beta} \\& \quad\leq{J_{3}} \Vert {A+B} \Vert _{\beta} \biggl( \frac {1}{2}+J_{31} \biggr) \Vert {F_{1}-F_{2}} \Vert _{\beta} +J_{3} \Vert {1+A} \Vert _{\beta} \Vert {F_{1}-F_{2}} \Vert _{\beta} \\& \quad\leq\biggl(J_{3} \biggl(J_{32}+ \frac{1}{2} \biggr) \Vert {A+B} \Vert _{\beta }+J_{3} \Vert {1+A} \Vert _{\beta} \biggr) \Vert {F_{1}-F_{2}} \Vert _{\beta} \\& \quad=\gamma \Vert {F_{1}-F_{2}} \Vert _{\beta}. \end{aligned}$$

 □

There is only one solution to the equation $NF = F $ by the compression mapping principle. So there is a unique solution to the linear boundary value problem *SR*, and the integral expression of the solution is (8).

## Conclusions

In this paper, we prove the existence of the solution to the nonlinear boundary value problem for the hypergenic function vector by virtue of the Arzela–Ascoli theorem and prove the existence and uniqueness of the solution to the linear boundary value problem for the hypergenic function vector by the compression mapping principle.
